# Axonal and dendritic localization of mRNAs for glycogen-metabolizing enzymes in cultured rodent neurons

**DOI:** 10.1186/1471-2202-15-70

**Published:** 2014-06-04

**Authors:** Brigitte Pfeiffer-Guglielmi, Benjamin Dombert, Sibylle Jablonka, Vanessa Hausherr, Christoph van Thriel, Nicole Schöbel, Ralf-Peter Jansen

**Affiliations:** 1 Interfaculty Institute for Biochemistry, University of Tübingen, Hoppe-Seyler-Str. 4, D-72076 Tübingen, Germany; 2 Institute for Clinical Neurobiology, University of Würzburg, Würzburg, Germany; 3 Leibniz Research Center for Working Environment and Human Factors, Dortmund, Germany; 4 Department of Cell Physiology, University of Bochum, Bochum, Germany

**Keywords:** Energy metabolism, Fluorescence *in-situ* hybridization, Glycogen phosphorylase, Glycogen synthase, mRNA localization, Neuronal primary culture

## Abstract

**Background:**

Localization of mRNAs encoding cytoskeletal or signaling proteins to neuronal processes is known to contribute to axon growth, synaptic differentiation and plasticity. In addition, a still increasing spectrum of mRNAs has been demonstrated to be localized under different conditions and developing stages thus reflecting a highly regulated mechanism and a role of mRNA localization in a broad range of cellular processes.

**Results:**

Applying fluorescence *in-situ*-hybridization with specific riboprobes on cultured neurons and nervous tissue sections, we investigated whether the mRNAs for two metabolic enzymes, namely glycogen synthase (GS) and glycogen phosphorylase (GP), the key enzymes of glycogen metabolism, may also be targeted to neuronal processes. If it were so, this might contribute to clarify the so far enigmatic role of neuronal glycogen. We found that the mRNAs for both enzymes are localized to axonal and dendritic processes in cultured lumbar spinal motoneurons, but not in cultured trigeminal neurons. In cultured cortical neurons which do not store glycogen but nevertheless express glycogen synthase, the GS mRNA is also subject to axonal and dendritic localization. In spinal motoneurons and trigeminal neurons *in situ*, however, the mRNAs could only be demonstrated in the neuronal somata but not in the nerves.

**Conclusions:**

We could demonstrate that the mRNAs for major enzymes of neural energy metabolism can be localized to neuronal processes. The heterogeneous pattern of mRNA localization in different culture types and developmental stages stresses that mRNA localization is a versatile mechanism for the fine-tuning of cellular events. Our findings suggest that mRNA localization for enzymes of glycogen metabolism could allow adaptation to spatial and temporal energy demands in neuronal events like growth, repair and synaptic transmission.

## Background

Neurons are highly polarized cells and mRNA localization to dendritic and axonal processes and local protein synthesis provide a means for the spatial and temporal fine-tuning of cellular events. Triggered by extrinsic signals, local translation of mRNAs can mediate growth, navigation and synapse formation in growing axons and dendrites as well as metabolic maintenance and repair in mature cells (for overview, see [1,2]). Studies with injured and uninjured axons of CNS neuronal cultures revealed the prevalence of mRNAs related to axonal guidance and synaptic function in regenerating neurons, while those for components of intracellular transport, mitochondria and cytoskeleton were more abundant in uninjured neurons [3]. In axons from explant cultures of embryonic and adult sensory neurons, microarray analysis revealed a repertoire of about 3000 localized mRNAs which changed significantly during development from embryonic to adult [4]. In cultures of rat sympathetic neurons, the mRNA for Impa1, a key-enzyme of the inositol signaling pathway, was identified as the most abundant transcript in axons [5]. The spectrum of mRNAs localized to axons also comprises mRNAs encoding enzymes of energy and carbohydrate metabolism, e.g. enolase, phosphoglycerate kinase and glucose-6-phosphate dehydrogenase [4], indicating a functional role of mRNA localization also in basic metabolic pathways. Among the mRNAs present only in axons of embryonic cultures were also those for glycogenin 1 and the brain isoform of glycogen phosphorylase [4].

Glycogen represents the major brain energy reserve which is located mainly in astrocytes [6]. Though its precise functions are still under debate, it has been proposed to be an emergency fuel store during physiological and pathological stress such as hypoglycemia and cerebral ischemia [7-9], but there is evidence for a role of glycogen also in normal metabolism, e.g. in learning and memory [10]. In the astrocyte-neuron lactate shuttle hypothesis (ANLSH), lactate derived from astrocytic glycogen and trafficking to adjacent neurons has been attributed a major role in brain energy metabolism [11]. In contrast to the CNS, the role of glycogen in the PNS has only recently been studied [12].

Because of the metabolic instability of glycogen, the presence of the key enzymes of glycogen metabolism, glycogen phosphorylase (GP) and glycogen synthase (GS), could serve as an indicator for glycogen, though this does not necessarily prove its presence. Applying GP isozyme-specific antibodies on rat tissue sections, it could be demonstrated that astrocytes express the muscle (MM) as well as the brain (BB) isozyme of glycogen phosphorylase while cortical neurons are devoid of immunoreactivity for both isoforms [13]. Neurons of the PNS, however, but also large motoneurons of the spinal cord express the BB isoform only [13-15]. Remarkably, GPBB is not only present in the cell soma, but also in the axons of spinal and vagus nerves, proposing a special role for glycogen in peripheral nerves. The presence of GP protein in peripheral axons with their appreciable length raises the question for a possible trafficking of its mRNA instead of the transport of the protein. This would be favorable, because it would endow the axon with the autonomy for local GP synthesis and thereby meet the special energy needs e.g. in growing and regenerating axons.

To study a possible axonal and dendritic localization of the mRNAs for GP and GS, we visualized the mRNAs with fluorescence *in-situ* hybridization (FISH) on three types of cultured neurons: Spinal motoneurons (motoneuron culture, MNC), cortical neurons (neuronal primary culture, NPC) and trigeminal neurons (trigeminal neuron culture, TNC). To compare FISH results on cultured cells with mRNA distribution patterns *in situ*, FISH was also applied on tissue sections. To investigate whether, in addition to GS, GP is also expressed in cortical neurons, the expression rates of GP mRNA and protein in NPC were determined by qRT-PCR and Western blot analysis.

## Methods

All experiments involving animals were carried out according to the law of animal experimentation issued by the German parliament (“Tierschutzgesetz”) and to the European Communities Council Directive. The present study was approved on May 16 2012, by the *Regierungspräsidum Tübingen (Baden-Württemberg, Germany)* according to § 4, Abs.3 of the law of animal experimentation.

### Cell cultures

For all experiments, we used standard protocols established and routinely applied in our labs. All cultures were immunocytochemically characterized using established markers.

NPC were prepared from ED 16 Wistar rat brains. Shortly, brains were dissected from the embryos and collected in Hibernate-E Medium (life technologies, Darmstadt, Germany) supplemented with B27 supplement and GlutaMAX (life technologies). Brains were mechanically dissociated by passing them through a nylon cloth of 110 μm mesh size. After centrifugation at 400 g and 4°C for 10 min, the cell pellet was resuspended in Neurobasal Medium (life technologies) supplemented as above. The cell suspension was passed through a second nylon cloth of 25 μm mesh size, appropriately diluted and seeded at a density of 1–3 million cells/ 21 cm^2^ surface area in 5 ml medium on p-D-lysine-coated coverslips. Cells were cultured at 37°C and 5% CO_2_ in a humidified atmosphere. For all experiments, 4–7 DIV cultures were used. Astroglial primary cultures (APC) were prepared from newborn Wistar rat brains as described [16]. For isolation of RNA and preparation of homogenates for Western blotting, 10 DIV cultures were used.

Studies on GP had primarily been carried out with rat tissues and primary cultures derived from rat brain, but studies carried out with tissues and cultures from other species confirmed the results [17,18]. We therefore used mouse spinal motoneuron and trigeminal neuron cultures for FISH analysis in motoneurons and trigeminal neurons. MNC were prepared from the lumbar spinal cords of ED 13.5 Bl/6 mice as described [19] and used for experiments after 5 DIV. TGC were prepared from the trigeminal ganglia of PD 0–5 or ED 16.5 CD1 mice as described [20] and used for experiments after 3–7 DIV.

### Tissue sections and immunohistochemistry (IHC)

Tissues were freshly dissected from adult Wistar rats of both sexes (4 animals for spinal cord sections, 3 animals for trigeminal ganglion sections, 2 animals for ventral spinal nerve and trigeminal nerve sections, respectively) and fixed in 4% paraformaldehyde/phosphate-buffered saline (PFA/PBS) at 4°C for 24 h. After cryoprotection, the tissue was frozen by immersion in liquid nitrogen. Sections were cut at 10 μm and stored at -80°C until processed.

### Riboprobe preparation

Total RNA was isolated from whole rat brain using TRI Reagent Solution (Ambion, Life Technologies) according to the manufacturer’s protocol. About 1 μg of total RNA was reversely transcribed using Oligo(dT) primers and 2 μl of the resulting cDNA solution were used as a template for the PCR amplification of nucleotide sequences from the coding regions of rat glycogen synthase 1 (accession number BC 131849) and rat brain glycogen phosphorylase (accession number NM 0131188). The following primers were used: GS, forward primer 5′-AGCCATCTTTGCGACTCAGC-3′, reverse primer 5′-TGGTAGGACTCAGGGGCTCA-3′; GP, forward primer 5′-TCCCAGACAAGGTAGCCATC-3′, reverse primer 5′-AAGGCCTCATCATCAACCAG-3′. The PCR products were diluted 1:10 and used as templates for a second PCR amplification applying primers including sequences of the T7 and SP6 RNA polymerase promoters, respectively. Primers were the following: GS, forward primer SP6, 5′-ATTTAGGTGACACTATAGACACCCTCACTGTCTCGACAC-3′, reverse primer T7 5′- TAATACGACTCACTATAGGGTGTACTGAGTGAGCTGGAGG-3′; GP, forward primer SP6 5′-ATTTAGGTGACACTATAGATCCCAGACAAGGTA-3′, reverse primer, T7 TAATACGACTCACTATAGGAAGGCCTCATCATCACC-3′. The products from 5 PCRs were pooled and purified. For GS DNA, electrophoresis in a 1% agarose preparative gel with subsequent extraction using a gel extraction kit (QIAGEN, Stockach, Germany) according to the manufacturer’s protocol was applied; GP DNA was purified with a PCR purification kit (QIAGEN) according to the manufacturer’s protocol.

Digoxigenin(DIG)-labeled RNA probes were prepared by in-vitro transcription with T7 RNA polymerase (antisense probe) or SP6 RNA polymerase (sense probe), respectively, using a DIG RNA Labeling Kit (Roche, Mannheim, Germany) according to the manufacturer’s protocol. The length of the transcripts was checked by agarose gel electrophoresis, subsequent capillary transfer to a nylon membrane and visualization of the bands with an alkaline phosphatase-coupled DIG antibody and nitroblue tetrazolium chloride/5-bromo-4-chloro-indolylphosphate substrate reaction. The lengths of the riboprobes were approximately 250 nt for GS and 600 nt for GP. Dot blots were used for semi-quantitative analysis of the probe concentrations.

### Fluorescence *in-situ* hybridization

After defined periods of time in culture, cells were fixed in 4% PFA/PBS at 4°C for 10 min, then washed with PBS and subsequently permeabilized with PBS/0.3% Triton X-100 at RT for 10 min. After a washing step in PBS, cells were pre-hybridized at 55°C for 0.5 – 2 h in the following hybridization buffer: 4x saline-sodium citrate (SSC), 4x Denhardt’s, 10% dextrane sulfate, 500 μg/ml salmon sperm DNA, 250 μg yeast tRNA and 50% formamide. Probes were denatured at 90°C for 5 min. Hybridization was performed in hybridization buffer at 55°C overnight with a probe concentration of 5 ng/μl for MNC and 0.1 ng/μl for the other cultures. Stringency washes were performed with 0.2x SSC at 55°C for MNC and 72°C for other culture types for 1 h and then with 0.2x SSC at room temperature (RT) for 5 min. After a blocking step in 0.5% Blocking Reagent (Roche) at RT for 30 min, cells were incubated with sheep anti-DIG peroxidase (POD) conjugate (F_ab_-fragments, Roche) diluted 1:500 in blocking reagent at RT for 3 h. After two washing steps in 0.1 M Tris/HCl, 0.15 M NaCl pH 7.5, 0,05% Tween 20 cells were incubated with the TSA-Plus Fluorescein reagent (Perkin Elmer, Rodgau-Jügersheim, Germany), diluted 1:50 (MNC) or 1:100 (other cell types) for 10 min (MNC) or 30 min (other cell types). After two washing steps, cells were rinsed with water and embedded in Immumount. Negative controls included replacement of antisense probe by sense probe, omitting of probes and omitting of anti-DIG antibody. In experiments combining FISH with immunocytochemistry (ICC), the relevant primary antibody was incubated together with the anti-DIG/POD conjugate. After the washing steps, cells were incubated with the secondary antibody diluted 1: 1000 at RT for 1 h. For FISH on tissue slices, the protocol was essentially the same with the following exceptions: Sections were postfixed in ice-cold 4% PFA/PBS for 5 min, and after stringency washes, endogenous peroxidase was blocked by incubation in 2% H_2_O_2_/1x SSC at RT for 15 min. Images were acquired with a Zeiss Cell Observer microscope using a 40x oil immersion objective and AxioVision software. Immunostaining on tissue sections and NPC that was not combined with FISH was performed as described [13], with the exception that secondary antibodies were conjugated to Alexa Dye 488 or 568 and diluted 1:1000. For TGC, the staining protocol was essentially identical. In brief, unspecific binding was blocked by incubation in 5% donkey serum/PBS for 30 min prior to incubation with antisera. All antibodies were diluted in 1% donkey serum; GP immunostaining was combined with staining for β-tubulin (1:2000) at RT for 2 h. Secondary antibodies were conjugated to Alexa dye 488 (anti-rabbit) or Cy5 (anti-mouse), diluted 1:500 and applied at RT for 30 min. Nuclei were stained with 4′,6-diamidino-2-indole (DAPI) diluted 1:10 000 in PBS .

### qRT-PCR

Total RNA was isolated from NPC, APC, rat cortex and cerebellum using TRI-Reagent (Ambion) according to the manufacturer’s protocol. After DNase treatment (RQ1 DNase, Promega, Mannheim, Germany), 250 ng of RNA were reversely transcribed applying the High Capacity cDNA Reverse Transcription Kit (Applied Biosystems, Life Technologies). For PCR reactions, 3.1 ng of cDNA were used. Reactions were prepared with Fast SYBR Mix (Applied Biosystems) and the following primers: GP, forward primer 5′- TCAGGGATGTAGCCAAGGTC-3′, reverse primer 5′-TCGGTTGTACAGGGTGATGA-3′; GS, forward primer 5′-CCCCCAATGGACTAAATGTG-3′, reverse primer 5′-AGCGTCCAGCGATAAAGAAA-3′; GFAP, forward primer 5′- GAAGAAAACCGCATCACCAT-3′, reverse primer 5′-TCCTTAATGACCTCGCCATC-3′; GAPDH, forward primer 5′-CTCATGACCACAGTCCATGC-3′, reverse primer 5′-TTCAGCTCTGGGATGACCTT-3′; β-actin, forward primer 5′-AGCCATGTACGTAGCCATCC-3′, reverse primer 5′-ACCCTCATAGATGGGCACAG-3′; 18S rRNA, forward primer 5′-GCAATTATTCCCCATGAACG-3′, reverse primer 5′-GGCCTCACTAAACCATCCAA-3′. All PCR reactions were performed in triplicate and repeated at least 6 times. Each experiment included a no-reverse transcription control and a no-template control. Amplification efficiencies were tested for each gene and reached optimal values (>1.8). C_t_ values were normalized to GAPDH, β-actin and 18S rRNA. Expression rates were expressed as n-fold of the expression rate of the relevant gene in APC.

### Western blot analysis

Western blotting was performed as described [13] with the following exceptions: proteins were transferred to a PVDF membrane using a semi-dry blotting system (BioRad, München, Germany). After a blocking step, the membrane was incubated with goat anti-rabbit IgG/POD conjugate (diluted 1:10 000) at RT for 2 h. The blot was developed applying a chemiluminescence detection system (ECL Western Blotting Substrate (Pierce, Rockford, IL, USA).

### Antibodies

Rabbit antiserum against rat brain glycogen phosphorylase isozyme was generated as published [13]. Anti-MAP2 monoclonal antibody was purchased from Millipore (Darmstadt, Germany). Rabbit antiserum against tau and monoclonal antibodies against GFAP and GAP-43 were from Sigma/Aldrich (St. Louis, MO, USA). Donkey antiserum against β-tubulin was purchased from Covance (Princeton, NJ, USA). Fluorescent secondary antibodies were from Molecular probes (Invitrogen), goat anti-rabbit POD conjugate was from Jackson Immuno Research (Dianova, Hamburg, Germany).

## Results

### GP and GS FISH on cultured lumbar motoneurons

**Figure 1:**
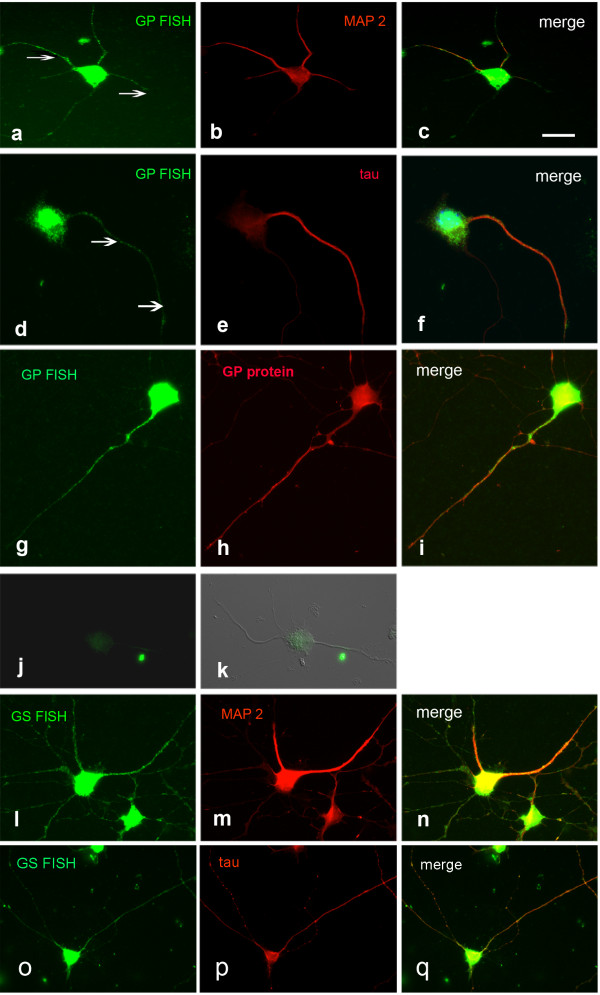
**FISH detection of GP and GS mRNA in spinal motoneurons, 5 DIV.** GP mRNA colocalizes with MAP 2 **(a-c)** and tau **(d-f)**. Arrows in **a** and **d** point to mRNA localized to neuronal processes. The signals for GP mRNA and protein are congruent **(g-i)**. **j**, **k** Negative control applying GP sense probe instead of antisense. **j** FISH, **k** FISH combined with Nomarski optics. GS mRNA also colocalizes with MAP 2 **(l-n)** and tau **(o-q)**. Bar in **a** = 20 μm and applies to **a**-**q**.

To study the mRNA expression of GP and GS, the key enzymes of glycogen metabolism, we applied FISH on cultured spinal motoneurons. This also allowed us to investigate the distribution of these mRNAs in soma, dendrites and axons. GP mRNA was present in the cell soma as well as in processes (Figure 1a, d, arrows). The signal overlapped with the signal for MAP2 (Figure 1b,c) as well as that for tau (Figure 1e, f) indicating the presence of the mRNA in dendrites as well as in axons. GP protein was also detectable in somata and processes (Figure 1g-i). As a specific control, applying GP sense probe instead of antisense resulted in only minimal color development (Figure 1j, k). GS mRNA was also localized to the processes and matched the patterns of MAP2 (Figure 1l-n) and tau (Figure 1o-q).

### GP FISH and IHC on spinal cord sections

**Figure 2:**
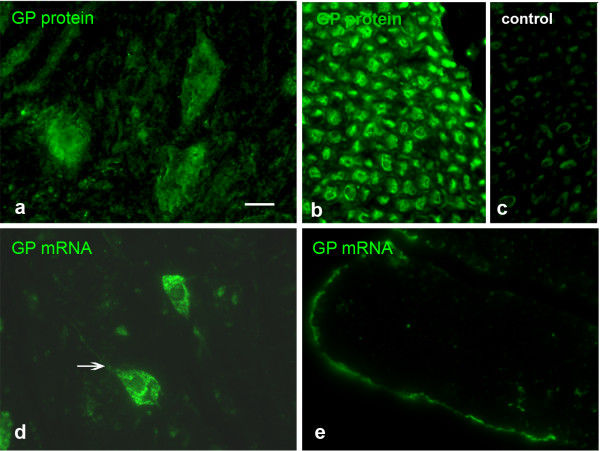
**GP IHC and FISH applied on sections of lumbar spinal cord and ventral spinal nerve.** GP protein is prominent in large motoneurons **(a)** and the axons of the ventral root of the spinal nerve **(b)**. Occasional signal can be detected in Schwann cells, but this is also the case in the negative control applying non-immune serum instead of immune serum **(c)**. GP mRNA is mainly present in the neuronal cell bodies **(d)**. No GP mRNA is present in the axons of the ventral spinal nerve **(e)**. The arrow in **d** points to mRNA in the initial segment of a process. Bar in **a** = 20 μm and applies to **a**-**e**.

We then went on to study the distribution of GP mRNA and protein *in vivo* in tissue sections. In cross-sections of lumbar spinal cord, GP protein as well as the mRNA were detectable in large motoneurons (Figure 2a,d); the mRNA signal was also visible in the proximal segments of thick processes (arrow Figure 2d). Interestingly, in cross-sections of the ventral spinal root, GP protein was abundant in the axons whereas no mRNA was detectable (Figure 2b, e). GS mRNA showed the same distribution pattern as GP mRNA (not shown).

### GS and GP FISH on cultured cortical neurons

**Figure 3:**
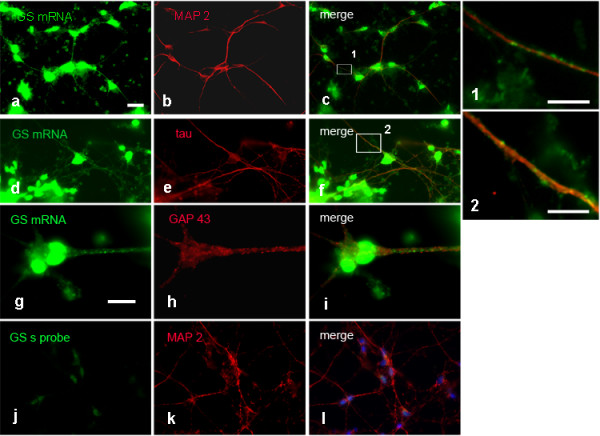
**FISH detection of GS mRNA in cortical neuronal cultures, 4 DIV.** GS mRNA is prominent in neuronal cell bodies and colocalizes with MAP 2 in dendrites **(a-c)** and tau in axons. **(d-f)**. Higher magnification (1 and 2) of the boxed areas in **c** and **f** reveals mRNA clusters in dendritic and axonal processes. FISH for GS mRNA combined with GAP 43 ICC reveals both signals in the axon, but colocalization in growth cones is not obvious **(g-i)**. **j**-**l** Negative control applying sense probe in combination with MAP 2 ICC. Bar in **a** = 20 μm and applies to **a-f** and **j-l**. Bar in **g** = 10 μm and applies to **g**-**i**. Bar in 1 = 5 μm, bar in 2 = 10 μm.

Based on the finding that GP and GS mRNAs are localized to the processes in cultured motoneurons, we then studied GS and GP mRNA expression in cultured cortical neurons. FISH experiments were performed on DIV 4 to DIV 7 cultures and displayed no culture time-dependent differences. GS mRNA was detectable in cultured cortical neurons and colocalized with the dendritic marker MAP2 (Figure 3a-c), and the axonal marker tau (Figure 3d-f). The signal was visible in the cell soma and the processes. Higher magnification (boxed areas 1 and 2 in c and f) revealed a granular appearance of mRNA in the processes (Figure 3d-f). As an additional neuronal marker, we used GAP 43 (Figure 3g-i). GS mRNA as well as GAP 43 were present in the axon, though direct overlap of the signals in growth cone-like structures was not obvious. Applying the sense probe resulted in a signal of neglectible intensity compared to that detected with the antisense probe (Figure 3j-l).

**Figure 4:**
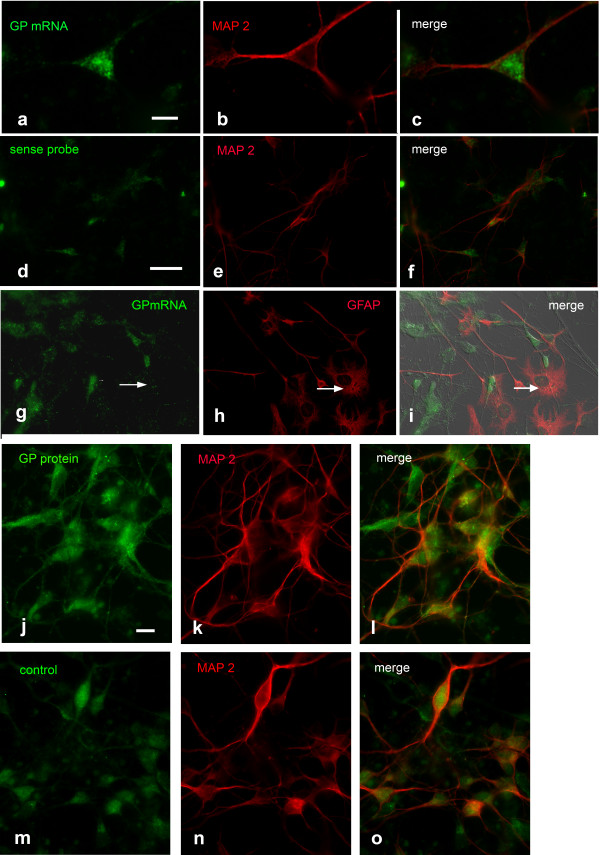
**Upper panel (a-i): Detection of GP mRNA in cortical neuronal cultures, 6 DIV.** Combination of GP FISH with MAP 2 ICC reveals a weak FISH signal **(a-c)**. **d**-**f** Negative control applying sense probe combined with MAP 2 ICC. **g**-**i** GP FISH combined with GFAP ICC. The arrows point to a GFAP positive astrocyte not expressing GP mRNA. Lower panel (j-o): Detection of GP protein in cortical neuronal cultures, 6 DIV. Double-labeling for GP with MAP 2 **(j-l)** reveals a weak signal for GP similar to that in the negative control **(m-o)**. Co-staining with rabbit normal serum in combination with MAP 2 antibodies indicates background staining level. Bar in **a** = 10 μm and applies to **a-c**, bar in **d** = 20 μm and applies to **d-i**, bar in **j** = 10 μm and applies to **j-o**.

In contrast to GS, the GP FISH signal was low and could only be detected in the soma and in the very proximal parts of neuronal processes (Figure 4a-c). The signal was higher in comparison to the signal level with the relevant sense probe (Figure 4d-f), but the difference was not significant enough to allow unambiguous conclusions. Astrocytes contaminating NPC did not express GP mRNA (arrows in Figure 4g-i). Immunocytochemical staining for GP protein in neuronal cultures revealed a weak signal (Figure 4j). However, the signal displayed when replacing GP antiserum by rabbit normal serum was not significantly lower (Figure 4m), suggesting that the detected signal was due to unspecific binding of the antibody. The results of FISH and ICC/IHC studies are summarized in Table 1.

**Table 1:** Summarized results of FISH and ICC/IHC studies on cultured neurons and tissue sections

	**GP mRNA**	**GS mRNA**	**GP protein**
MNC	soma and processes	soma and processes	soma and processes
motoneurons in spinal cord	positive	data not shown	positive
ventral root of spinal nerve	negative	data not shown	positive
NPC	low signal level, data not significant	soma and processes	low signal level, data not significant
TGC	soma	soma	soma and processes
neurons in trigeminal ganglion	positive	data not shown	positive
trigeminal nerve	negative	data not shown	positive

### qRT-PCR and Western blotting

**Figure 5:**
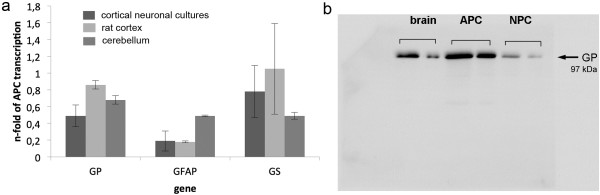
**qRT-PCR studies and Western blot analysis. a** GP, GS and GFAP mRNA expression levels were analyzed in cortical neuronal cultures, rat cortex and cerebellum. C_t_-values were normalized to GAPDH, β-actin and 18S rRNA. Expression rates of the genes were given as n-fold of the relevant rates in astroglial cultures. Data were shown as means ± SEM (n = 6). **b** GP protein Western blotting analysis in cortical neuronal cultures, whole rat brain and astroglial cultures. For each protein extract, 2,5 and 1 μg of protein were loaded.

Because the results from FISH as well as from immunocytochemistry concerning the presence of GP mRNA and protein in NPC were difficult to interpret, we performed qRT-PCR and Western blotting. We compared the amounts of GP and GS mRNA in NPC by qRT-PCR (Figure 5a) to those in brain regions and expressed them as n-fold of the mRNA present in astroglial cultures that are known to store glycogen and to express GP and GS. GP mRNA was clearly detected in NPC and reached 86% of the level found in cortex and 72% of the level found in cerebellum. GP mRNA in NPC mounted up to 62% of the transcript amount of GS and about 50% of that found in APC. Because of the possibility that GP mRNA in NPC might be due to the presence of contaminating astrocytes in NPC, glial fibrillary acidic protein (GFAP) mRNA expression was determined as an approximate measure for the percentage of astrocytes in the culture. Immunocytochemical analysis of NPC with GFAP antibody routinely reveals a percentage of 10–20% of contaminating astrocytes. GFAP mRNA expression in NPC was 0.19 fold of that of APC confirming the results of the immunocytochemical analysis. Western blotting clearly demonstrated the presence of GP protein in NPC (Figure 5b).

### GP FISH and IHC on cultured trigeminal neurons and trigeminal ganglion sections

**Figure 6:**
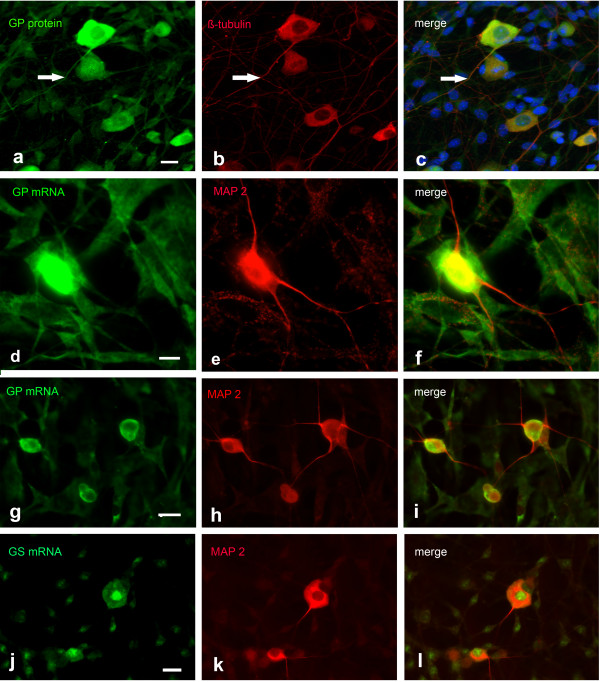
**FISH detection of GP and GS mRNA in cultured trigeminal neurons. a-c** ICC for GP combined with β-tubulin ICC. GP protein is present in neuronal cell bodies and processes (arrows in **a-c** point to a long neuronal process expressing GP). **d-f** FISH for GP combined with MAP 2 ICC, culture derived from postnatal trigeminal ganglia, 3 DIV. GP mRNA is abundant in neuronal cell bodies but absent from MAP 2 positive processes. **g-i** Same as in **d-f**, but cultures derived from embryonic ganglia, 2 DIV. GP mRNA is only present in neuronal cell bodies. **j-l** Combination of GS FISH with MAP 2 ICC, cultures derived from PD 2 ganglia. GS mRNA is only present in cell bodies and nuclear speckles **(j)**. Bar in **a** = 10 μm and applies to **a-c**, bar in **d** = 10 μm and applies to **d-f**, bar in **g** = 20 μm and applies to **g-i**, bar in **j** = 20 μm and applies to **j-l**.

**Figure 7:**
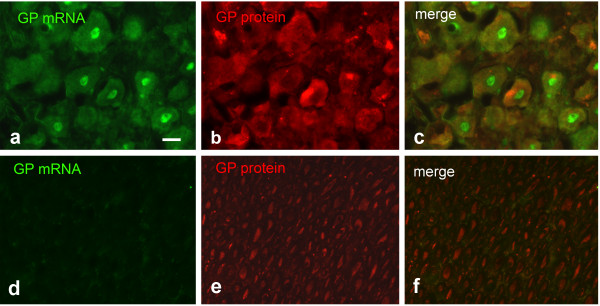
**GP FISH combined with GP IHC applied on sections of trigeminal ganglion and nerve. a-c** trigeminal ganglion, **d-f** trigeminal nerve. GP mRNA and protein is visible in the trigeminal cell bodies, trigeminal nerve shows no FISH signal but expresses GP protein. Bar in **a** = 20 μm and applies to **a-f**.

As a representative of peripheral sensory neurons, we investigated trigeminal neurons. These neurons expressed GP protein (Figure 6a-c). The signal was present in the cell soma. Especially in large neurons, the signal was also detectable in processes (arrows in Figure 6a-c). GP mRNA, however, was abundant in the cell soma but not detectable in the cell processes. This holds true for cultures derived from postnatal ganglia (Figure 6d-f) as well as for cultures derived from embryonic ganglia (Figure 6g-i). Likewise, GS mRNA is detectable in the soma but not in precesses (Figure 6j-l). In sections from adult trigeminal ganglia, GP mRNA and protein could be detected in neuronal soma (Figure 7a-c), whereas in trigeminal nerve the protein but not the mRNA was discovered (Figure 7d-f). The nuclear signal observed in Figure 6j for GS mRNA and in Figure 7a for GP mRNA is probably due to pre-mRNA in nuclear speckles.

## Discussion

mRNA localization to neuronal processes has primarily been described for proteins belonging to the categories of cytoskeletal and mitochondrial proteins, signaling molecules and nuclear factors. It was the aim of this study to investigate by FISH techniques whether the mRNAs for glycogen synthase and glycogen phosphorylase, the key players in glycogen metabolism and, consequently, major enzymes of energy metabolism are also transported to axons and dendrites. The occurrence of mRNA targeting in neurons could shed light on the still controversially discussed functions of glycogen in nervous tissues. The sites of glycogen synthesis, storage and metabolism have been under debate till now: In the CNS, glycogen is mainly stored in astrocytes [6,21] though under stressful conditions like trauma and fasting [6] or in hibernating animals [22] it could also be demonstrated in neurons. Likewise, the presence of glycogen-metabolizing enzymes in the CNS has been described in astrocytes [23-25] but also in cortical neurons [23] and in distinct types of neurons in the brain stem and spinal cord [13]. In contrast to the CNS, glycogen in the PNS has not been studied systematically. Recently, it has been demonstrated in Schwann cells of myelinated axons of the sciatic nerve [12]. GP immunoreactivity, however, was abundant in axons of peripheral nerves [15].

We found that GP and GS mRNAs were present in the cell soma of diverse cultured neurons but also in axonal and dendritic processes, indicating that the subcellular distribution of these mRNAs is regulated and that these mRNAs could also be translated in dendritic and axonal compartments. However, we observed major differences between different types of cell culture, which is in accordance with the significant variation between axonally localized mRNAs found in various transcriptome analyses, e.g. between intact and injured axons [3] or during development from embryonic to adult [4]. In cultured spinal cord motoneurons GP as well as GS mRNAs were localized even to far-distant regions of processes (Figure 1). In contrast, no localization was evident in cultured trigeminal neurons (Figure 6). Trigeminal neurons receive sensory input from the facial skin and mucosae and are analogous to DRG neurons that receive sensory input from the skin in limbs and trunk. DRG neurons as well as spinal nerve have been shown to express GP abundantly [15], and this is also the case in the trigeminal ganglion and nerve (Figure 7). Accordingly, trigeminal neurons contain GP protein in their processes and should therefore be a probable candidate for mRNA localization, which was not observable. This is in contrast to spinal cord motoneurons, which are known to express GP protein though they are part of the CNS. With their axons projecting to muscles, however, they are also part of the PNS, and GP could be demonstrated in axons of the ventral spinal nerves (Figure 2). With GP protein present and with respect to the appreciable length of trigeminal and spinal nerves, both types of neurons would be considered probable candidates for mRNA targeting to processes, but this was only the case for motoneurons. The difference might be attributed to several factors. First, there are principal differences between motoneurons and sensory neurons. Motoneurons but not trigeminal neurons are affected in severe diseases like spinal muscular atrophy (SMA, [26]) or amyotrophic lateral sclerosis (ALS, [27,28]). The vulnerability of these neurons might be due to the length of their axons, which implies transport over long distances. The molecular motors involved in these transport processes require ATP. Fast axonal transport is energized by glycolytic enzymes located on motile vesicles rather than by mitochondrially produced ATP [29]. Glycolysis might be fueled by glucose, which could be provided by a spatially controlled local GP translation and subsequent glycogen degradation. For those neurons, local GP and GS synthesis aided by GP and GS mRNA localization might be especially favorable because it would allow a fine-tuned energy supply. Second, the developmental states of neurons at the time point they were taken in culture are different and reflect variable metabolic requirements. In addition, culture conditions are not identical. For example, spinal motoneuron cultures are extremely pure cultures whereas TGC contain an appreciable fraction of non-neuronal cells like Schwann cells and fibroblasts, which contribute to growth and nourishment. Local glycogen degradation in processes might therefore be of lower importance. Third, mRNA localization depends on the interplay of diverse molecular motors [30] with the superfamily of kinesin proteins playing an important role [31]. Different types of kinesins are expressed in different types of neurons, and lack of the correct kinesin might also account for the absence of GP and GS mRNA localization in TGC.

Though GP and GS mRNAs are localized to processes in spinal motoneurons under culture conditions, this could not be demonstrated *in situ* by applying FISH on tissue sections (Figure 2). This is not necessarily a discrepancy, because neurons in culture encounter an artificial environment and are subject to factors and molecules quite different from those *in vivo*. Also, mRNA localization plays a major role in developmental processes like neuronal maturation and axonal outgrowth (for overview, see [32]) implying a versatile mRNA distribution. Gumy et al. reported on a dynamical change of localized mRNA repertoires in explants cultures from embryonic to adult [4]. Among the mRNAs only localized to axons in cultures derived from embryonic tissues were also those for GPBB and glycogenin, the self-glycosylating activity of glycogen synthase. In addition, neurons in culture share properties with regenerating neurons, which have mRNA spectra different from uninjured cells [3]. Trigeminal neurons do not localize GP/GS mRNA to their processes, neither in culture nor *in situ*. Whether conditions could be found which would allow targeting, must be subject of extended investigations.

Cortical neurons do not store glycogen but express GS [23]. The enzyme is locked in an enzymatically inactive state by an intricate enzymatic machinery. Failure of its inhibition results in a severe neurological disorder named Lafora disease that is characterized by the accumulation of an atypical toxic glycogen [33]. GS mRNA has been demonstrated in NPC by Northern blotting [34]. In our studies, FISH demonstrated GS mRNA presence in the neuronal somata as well as in the processes of cultured cortical neurons. This localization points to a function of GS apart from synthesizing glycogen. In HeLa cell ribosomes, GS has been shown to be involved in translational control [35]. Non-enzymatic functions for enzymes, a phenomenon called moonlighting, have been described for many enzymes, among them glycolytic ones [36]. Among those is another enzyme of the basic carbohydrate metabolism, GAPDH, for which evidence suggests a role in the fusion of vesicles to membranes [37]. In contrast to GS, GP and its mRNA has previously not been demonstrated in NPC [25,34]. This might be attributed to technical reasons like low sensitivity of the methods applied. Therefore, we used qRT-PCR and Western blotting with chemiluminescence detection in addition to FISH and ICC. The latter methods did not lead to unequivocal results because the signal intensity did not differ significantly from that reached in the negative control experiments. qRT-PCR and Western blotting, however, revealed the presence of GP mRNA and protein in NPC. GP mRNA in NPC might be attributed to contaminating astrocytes which, according to immunocytochemical analysis and GFAP mRNA expression, can mount up to about 20%. These astrocytes, however,are not a probable source for GP mRNA and protein because they do not express GP mRNA (Figure 4g-i) and do not stain for GP protein [25]. This might be due to the fact that for astrocyte primary cultures, postnatal brains were used while neuronal cultures were derived from embryonic brains. Astrocytes in the latter culture might therefore be in a different state of differentiation. Also, the presence of certain neurons selected for by the culture conditions might influence the expression of GP. Western blotting demonstrated the presence of GP protein in NPC, though in smaller amounts compared to APC.

The question whether cortical neurons do express GP could not be answered definitely by our investigations. However, potent qRT-PCR analysis pleads in favor of GP mRNA expression, and GP should be thought of as a possible additional player, though with a still enigmatic role.

## Conclusions

We could show that the mRNAs for GS and GP, two major enzymes of the metabolism of glycogen, the single large energy reserve in neural tissues, can be localized to neuronal processes. This might have important consequences for neuronal functionality under energy demanding conditions like growing, repair and memory consolidation. Because cultured spinal motoneurons were the most impressive example for GS and GP mRNA localization, a so far unknown role for glycogen in these neurons with their far-reaching processes should be discussed. In contrast to peripheral neurons, cortical neurons do not store glycogen under non-pathological conditions but express GS, and, as shown in our studies, do localize its mRNA to neuronal processes. This may indicate a moonlighting function for GS.

The results of our investigations are another jigsaw in the glycogen puzzle. To approach the functional role(s) of glycogen in peripheral neurons, metabolic studies are now of high importance.

## Abbreviations

GS: Glycogen synthase; GP: Glycogen phosphorylase; FISH: Fluorescence *in-situ* hybridization; MNC: Motoneuron culture; NPC: Neuronal primary culture; TNC: Trigeminal neuron culture; APC: Astroglial primary culture; DIV: Days *in vitro*; IHC: Immunohistochemistry; PFA/PBS: Paraformaldehyde/phosphate-buffered saline; DIG: Digoxigenin; POD: Peroxidase; ICC: Immunocytochemistry; DAPI: 4′,6-diamidino-2-indole; GFAP: Glial fibrillary acidic protein.

## Competing interests

The authors declare that they have no competing interests.

## Author’s contributions

BD and SJ carried out and financed motoneuron cultures. NS and CvT carried out and financed trigeminal neuron cultures. VH participated in the preparation of the latter and performed the relevant ICC experiments on these cultures. BPG designed the experiments, carried out cortical neuronal and astroglial cultures, FISH, ICC and IHC experiments and analyzed the data. RPJ financed the study and participated in data interpretation. The manuscript was written by BPG and RPJ. All authors read and approved the final manuscript.
